# Microbubbles decorated with dendronized magnetic nanoparticles for biomedical imaging: effective stabilization via fluorous interactions

**DOI:** 10.3762/bjnano.10.205

**Published:** 2019-10-31

**Authors:** Da Shi, Justine Wallyn, Dinh-Vu Nguyen, Francis Perton, Delphine Felder-Flesch, Sylvie Bégin-Colin, Mounir Maaloum, Marie Pierre Krafft

**Affiliations:** 1 Institut Charles Sadron (CNRS), University of Strasbourg, 23 rue du Loess, 67034 Strasbourg, France; 2 Institut de Physique et de Chimie des Matériaux de Strasbourg (IPCMS), University of Strasbourg, 23 rue du Loess, 67034 Strasbourg, France

**Keywords:** diagnostic imaging, fluorinated dendrons, fluorocarbon, iron oxide nanoparticles, magnetic nanoparticles, microbubbles

## Abstract

Dendrons fitted with three oligo(ethylene glycol) (OEG) chains, one of which contains a fluorinated or hydrogenated end group and bears a bisphosphonate polar head (C*_n_*X_2_*_n_*_+1_OEG_8_Den, X = F or H; *n* = 2 or 4), were synthesized and grafted on the surface of iron oxide nanoparticles (IONPs) for microbubble-mediated imaging and therapeutic purposes. The size and stability of the dendronized IONPs (IONP@C*_n_*X_2_*_n_*_+1_OEG_8_Den) in aqueous dispersions were monitored by dynamic light scattering. The investigation of the spontaneous adsorption of IONP@C*_n_*X_2_*_n_*_+1_OEG_8_Den at the interface between air or air saturated with perfluorohexane and an aqueous phase establishes that exposure to the fluorocarbon gas markedly increases the rate of adsorption of the dendronized IONPs to the gas/water interface and decreases the equilibrium interfacial tension. This suggests that fluorous interactions are at play between the supernatant fluorocarbon gas and the fluorinated end groups of the dendrons. Furthermore, small perfluorohexane-stabilized microbubbles (MBs) with a dipalmitoylphosphatidylcholine (DPPC) shell that incorporates IONP@C*_n_*X_2_*_n_*_+1_OEG_8_Den (DPPC/Fe molar ratio 28:1) were prepared and subsequently characterized using both optical microscopy and an acoustical method of size determination. The dendrons fitted with fluorinated end groups lead to smaller and more stable MBs than those fitted with hydrogenated groups. The most effective result is already obtained with C_2_F_5_, for which MBs of ≈1.0 μm in radius reach a half-life of ≈6.0 h. An atomic force microscopy investigation of spin-coated mixed films of DPPC/IONP@C_2_X_5_OEG_8_Den combinations (molar ratio 28:1) shows that the IONPs grafted with the fluorinated dendrons are located within the phospholipid film, while those grafted with the hydrocarbon dendrons are located at the surface of the phospholipid film.

## Introduction

Microbubbles (MBs), that is, micrometer-sized gas particles dispersed in an aqueous medium, are clinically used as contrast agents for ultrasound imaging, including molecular imaging, and actively investigated for surgical ablation, targeted drug and gene delivery [1–10]. They are also being examined for use, in conjunction with focused ultrasound, and under magnetic resonance imaging guidance, for achieving blood/brain and blood/tumor barrier crossing of drugs [11–12]. Medical MBs have a shell consisting of surfactants, phospholipids, or polymers and are usually stabilized by a fluorocarbon gas [13] that acts as an osmotic agent [14–15] and as a co-surfactant to phospholipids [16] and block co-polymers [17].

Nanoparticles can be attached to the bubble shells to extend their diagnostic and therapeutic potential by combining multimodal imaging, drug or gene delivery, and/or enhancement and control of the acoustic signal for energy deposition, as is required for sonothrombolysis or ablation surgery. MBs incorporating iron oxide nanoparticles (IONPs) are sought after as dual contrast agents for ultrasound and magnetic resonance imaging [18–20] and drug delivery [21–22]. The shells of the presently available MBs that incorporate IONPs are often made of polymers. For example, ultrasmall superparamagnetic iron oxide nanoparticles were embedded in the wall of poly(butyl cyanoacrylate)-based MBs, allowing the blood‒brain barrier penetration to be monitored [23]. Soft-shell colloids called lipospheres have also been reported for enhanced gene and drug delivery [24]. These lipospheres consist of gas-filled spheres coated by a film of soybean oil that encases the cargo of nanoparticles and is itself contained within a film of phospholipids [24]. Both polymer-shelled MBs and lipospheres have some advantages and some limitations [25]. In both cases, the shells can be custom-made to enhance stability, circulation duration, drug-loading capacity and release rate, targeting the fusion with cell membranes [24]. Both types of constructs are generally more stable, but less echogenic than “true” gas microbubbles, due to the dampening effect of the polymer shell or oil contained in the phospholipid coating [24–25]. One important difficulty encountered in the preparation of such magnetic MBs is that IONPs rapidly aggregate in aqueous media [25]. Commercially available 50 nm magnetic IONPs coated with phospholipids allowed for the preparation of MBs that enabled transfection of neuroblastoma cells with a generic, fluorescent, small, interfering RNA under magnetic and ultrasound fields [26].

In the present work, we incorporated IONPs coated by dendritic phosphonates bearing oligo(ethylene glycol) (OEG) chains into the phospholipid shell of the MBs. OEG chains were selected in order to improve the dispersibility of the IONPs in water [27–28]. Dendritic phosphonates are effective anchoring agents due to the covalent PO–metal bonds that stabilize aqueous dispersions of IONPs [27,29]. Such dendronized IONPs have been investigated for hyperthermia and magnetic resonance imaging owing to their increased stability in aqueous media and biocompatibility [27–28]. An even stronger anchoring agent consisting of a dendron structure bearing a bisphosphonate polar head provided increased colloidal stability in physiological media [30]. To the best of our knowledge, the implementation of dendronized IONPs in phospholipid-shelled MBs has not yet been reported. This approach is expected to combine some advantages over existing methods, including the ability to graft isolated IONPs instead of clusters at the MB surface, and allowing the microbubbles to go undetected, thus potentially minimizing the recourse to pegylated lipids.

**Figure 1:**
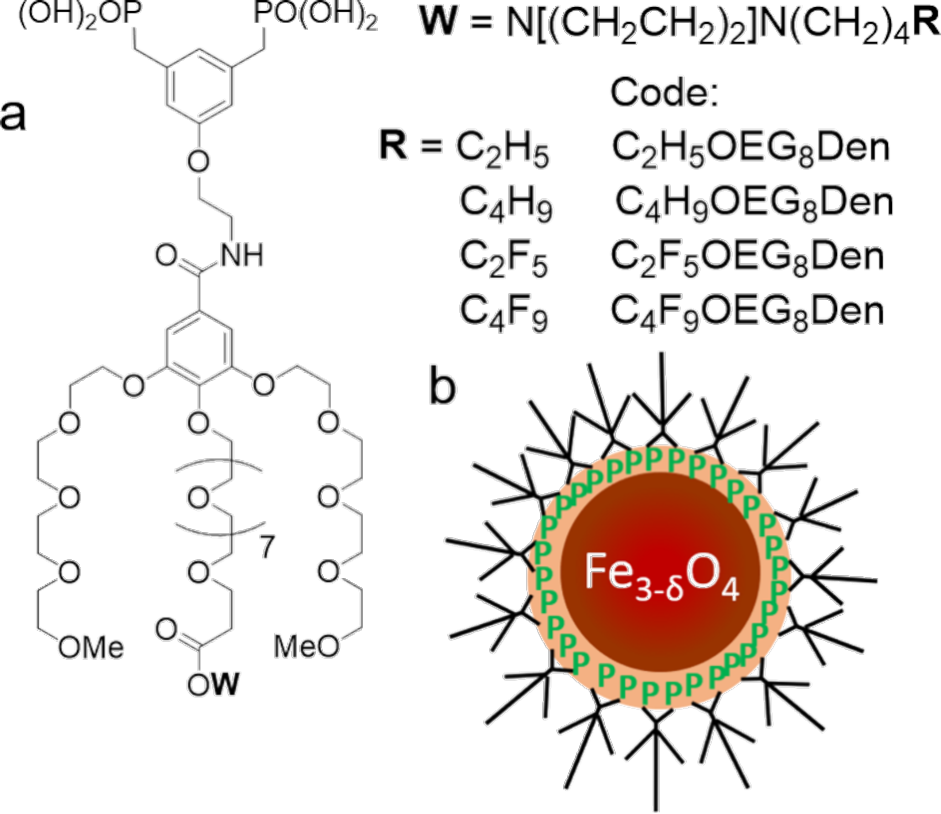
a) Molecular structure of the dendrons investigated (C*_n_*X_2_*_n_*_+1_OEG_8_Den, X = F or H; *n* = 2 and 4); b) Schematic representation of a dendronized IONP showing the anchoring of the bisphosphonate function on the iron oxide.

We report here the preparation of perfluorohexane (*F*-hexane)-stabilized MBs with a shell of dipalmitoylphosphatidylcholine (DPPC) that incorporates IONPs grafted with OEG bisphosphonate-headed dendrons. Four dendrons were synthesized and investigated that feature two phosphonic acids and three OEG chains, including a longer one in the para position. The latter was fitted with a fluorinated (C_2_F_5_ or C_4_F_9_) or a hydrogenated (C_2_H_5_ or C_4_H_9_) end group (C*_n_*X_2_*_n_*_+1_OEG_8_Den, X = F and H; *n* = 2 and 4, Figure 1). First, we present the synthesis and the characterization of the IONPs grafted with the selected dendrons (IONP@C*_n_*X_2_*_n_*_+1_OEG_8_Den). Second, we report the adsorption kinetics of IONP@C*_n_*X_2_*_n_*_+1_OEG_8_Den at the interface between air or *F*-hexane-saturated air and water. Third, we discuss the size and stability characteristics of *F*-hexane-stabilized DPPC-shelled MBs incorporating IONP@C*_n_*X_2_*_n_*_+1_OEG_8_Den. Fourth, we report an atomic force microscopy (AFM) study that reveals that the location of the dendronized nanoparticles in the phospholipid film strongly depends on the nature of the terminal group.

## Results and Discussion

### Synthesis and grafting of dendrons on iron oxide nanoparticles

**Figure 2:**
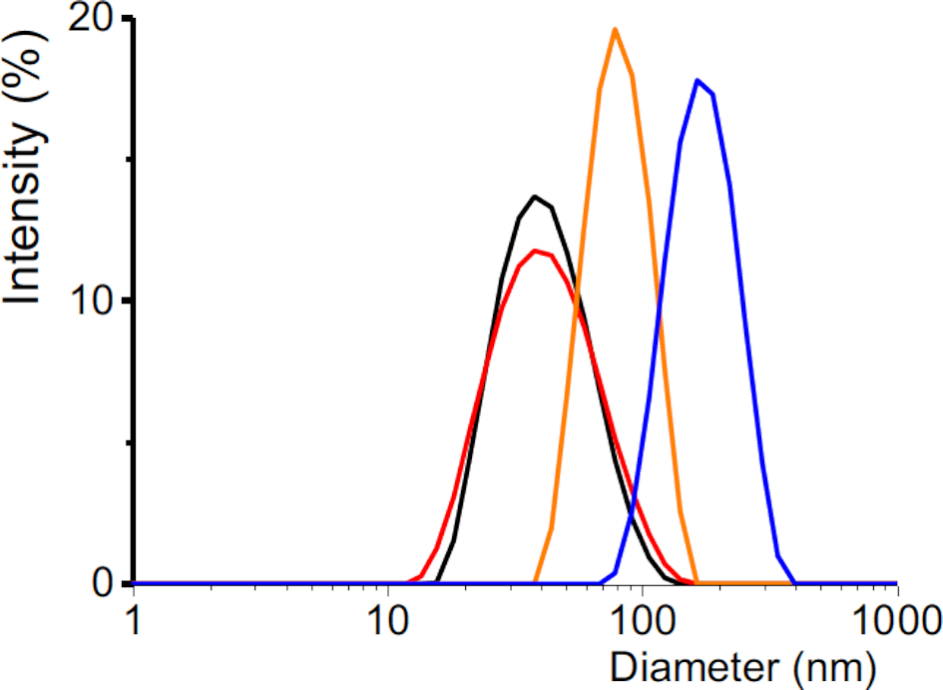
Hydrodynamic diameter distributions of IONPs grafted with dendrons: C_2_H_5_OEG_8_Den (38 ± 1 nm, black), C_2_F_5_OEG_8_Den (37 ± 1 nm, red), C_4_H_9_OEG_8_Den (95 ± 12 nm, orange), C_4_F_9_OEG_8_Den (197 ± 15 nm, blue) in aqueous dispersions (Fe conc. 0.05 mg mL^−1^).

The OEG dendrons were synthesized as described in the Experimental section. Briefly, the piperazine scaffold was selected as an appropriate template to introduce the perfluoroalkylated or alkylated chain on a generation 1 bisphosphonic dendron bearing three OEG chains [31]. In order to facilitate the insertion and increase the visibility of the perfluoroalkylated (or alkylated) end group in the microbubble wall, the central OEG chain carrying the piperazine moiety was lengthened (Figure 1a). A multistep chemical sequence allowed for the production of bisphosphonate dendrons at a reasonable yield (55–80%). IONPs (mean diameter of 9.0 ± 0.9 nm) were synthesized by thermal decomposition of iron (II) stearate in the presence of oleic acid in dioctyl ether, which enables better control of the size, morphology and composition of the IONPs [32]. The four dendrons were grafted on the magnetic IONPs by direct exchange of the ligand (oleic acid) according to [33]. The excess dendron material was removed by ultrafiltration. The grafting of the dendrons on the IONPs was assessed by infrared spectroscopy (IR), which showed a significant reduction of the oleic acid alkyl bands (2926‒2850 cm^−1^) and the appearance of the OEG signal (1096 cm^−1^) (Supporting Information File 1, Figure S1). The grafting of the dendrons on the IONPs was also confirmed by dynamic light scattering (DLS, Figure 2). The hydrodynamic mean diameter of IONP@C_2_X_5_OEG_8_Den (X = F or H) was ≈37 nm, which is significantly larger than the mean diameter of the oleic-acid-covered IONPs (≈10 nm, Supporting Information File 1, Figure S2). This can be ascribed to the fact that a corona of OEG chains is now present around the nanoparticle and captures molecules of water, which contributes to a further increase of the hydrodynamic radius. The mean diameter of the IONP@C_4_X_9_OEG_8_Den materials was larger, namely ≈95 nm and ≈200 nm for X = H and F, respectively, revealing that aggregation occurs in aqueous media due to the hydrophobicity of the end group. Fortunately, this did not preclude performing the adsorption kinetics studies. Altogether, owing to their dendritic structure, the OEG chains were found to confer excellent dispersibility and stability to the IONPs [33].

### Adsorption kinetics of dendronized nanoparticles at the gas/liquid interface

**Figure 3:**
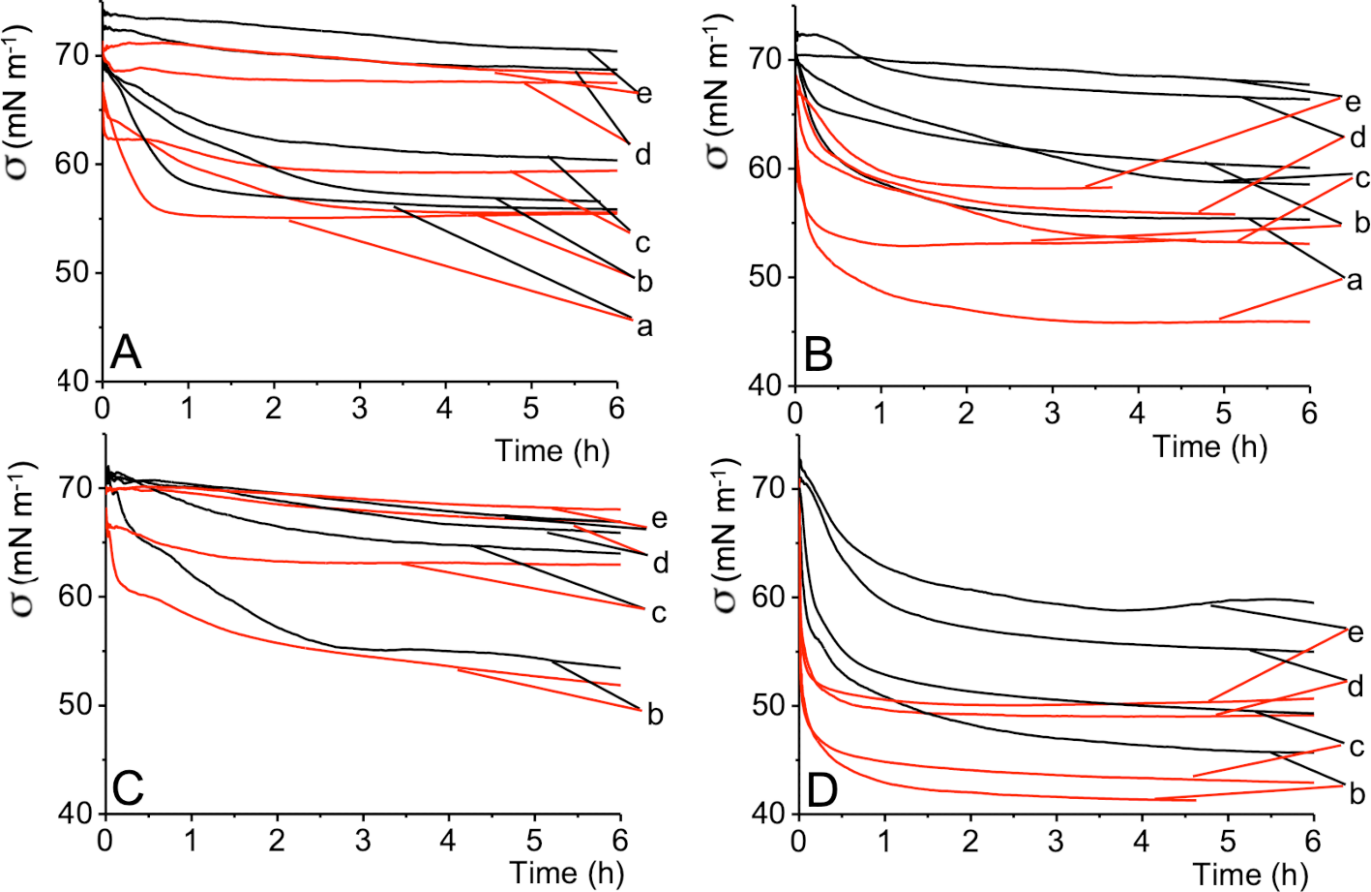
Adsorption kinetics of IONPs grafted with various dendrons measured at 25 °C: A) C_2_H_5_OEG_8_Den, B) C_2_F_5_OEG_8_Den, C) C_4_H_9_OEG_8_Den and D) C_4_F_9_OEG_8_Den, at various Fe concentrations: a) 0.1; b) 0.05; c) 10^−2^; d) 10^−3^ and e) 10^−4^ mg mL^−1^. The dendronized IONPs were exposed to air (black) or to *F*-hexane-saturated air (red).

The adsorption of the dendronized IONPs at the air/water and *F*-hexane-saturated air/water interface was first investigated using bubble profile analysis tensiometry. As described in our earlier reports [34–35], we first confirmed that *F*-hexane taken alone, when introduced into the gaseous phase of the tensiometer bubble, adsorbs rapidly onto the interface, as indicated by the instant reduction of the interfacial tension σ by ≈4 mN m^−1^ (from 72 to 68 ± 0.5 mN m^−1^, Supporting Information File 1, Figure S3). The concentration of Fe in the IONP dispersions was varied from 10^−4^ to 10^−1^ mol L^−1^. The variations of the interfacial tension σ over time are collected in Figure 3 and Table 1. The results show that, not surprisingly, σ decreases with increasing Fe concentration in all cases. The lowest σ values were obtained for the IONPs grafted with the fluorinated dendrons, reflecting their higher hydrophobicity. We also observed that exposure to *F*-hexane has two important consequences on the adsorption of the dendronized IONPs. First, the adsorption process is accelerated, and second, the equilibrium interfacial tensions are significantly lowered.

**Table 1:** Characteristic adsorption time τ (h) and interfacial tension at equilibrium σ_eq_ (mN m^−1^) of the IONPs grafted with hydrogenated or fluorinated dendrons.

	C_2_H_5_OEG_8_Den				C_2_F_5_OEG_8_Den				C_4_H_9_OEG_8_Den				C_4_F_9_OEG_8_Den			
	Air		*F*-hexane		Air		*F*-hexane		Air		*F*-hexane		Air		*F*-hexane	
Fe conc. (mol L^−1^)	τ	σ_eq_	τ	σ_eq_	τ	σ_eq_	τ	σ_eq_	τ	σ_eq_	τ	σ_eq_	τ	σ_eq_	τ	σ_eq_

1 × 10^−1^	0.58 ± 0.05	56 ± 1	0.28 ± 0.03	55 ± 2	0.56 ± 0.05	55 ± 1	0.17 ± 0.02	46 ± 1	–	–	–	–	–	–	–	–
5 × 10^−2^	1.36 ± 0.12	57 ± 2	1.11 ± 0.16	56 ± 2	1.11 ± 0.13	59 ± 2	0.56 ± 0.06	53 ± 1	1.25 ± 0.13	53 ± 2	1.11 ± 0.13	51 ± 1	0.28 ± 0.03	48 ± 2	0.03 ± 0.01	42 ± 2
1 × 10^−2^	1.31 ± 0.20	60 ± 1	0.56 ± 0.14	59 ± 1	2.22 ± 0.26	58 ± 1	0.89 ± 0.11	53 ± 1	2.22 ± 0.30	65 ± 1	1.06 ± 0.12	64 ± 1	0.33 ± 0.03	49 ± 2	0.03 ± 0.01	42 ± 1
1 × 10^−3^	2.08 ± 0.30	67 ± 3	0.42 ± 0.14	65 ± 1	2.22 ± 0.28	66 ± 1	0.56 ± 0.08	58 ± 2	8.61 ± 0.93	66 ± 1	4.72 ± 0.51	66 ± 2	0.92 ± 0.13	55 ± 1	0.06 ± 0.02	49 ± 1
1 × 10^−4^	18.61 ± 1.87	70 ± 2	4.44 ± 0.39	68 ± 2	38.89 ± 3.90	66 ± 2	0.75 ± 0.10	58 ± 2	10.28 ± 1.08	65 ± 2	7.50 ± 0.62	67 ± 2	0.78 ± 0.23	60 ± 2	0.03 ± 0.02	52 ±3

**Figure 4:**
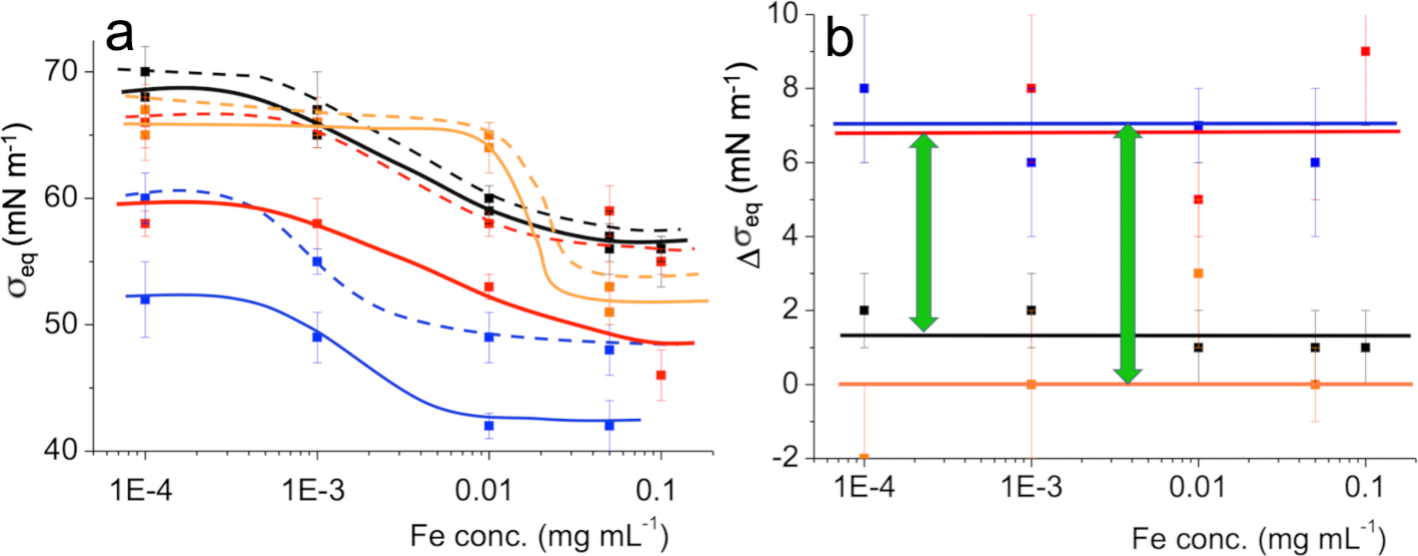
a) Variation of the interfacial tension at equilibrium (σ_eq_, 25 °C) as a function of the Fe concentration in dispersions of IONPs grafted with dendrons (C_2_H_5_OEG_8_Den: black; C_2_F_5_OEG_8_Den: red; C_4_H_9_OEG_8_Den: orange and C_4_F_9_OEG_8_Den: blue) at the air (dashed line) and *F*-hexane-saturated air (solid line)/aqueous phase interface. b) Variation of the difference of the interfacial tension at equilibrium (Δσ_eq_) measured under air and under *F*-hexane-saturated air versus Fe concentration for various IONPs (same color code). The green arrows highlight the difference in behavior between fluorinated and non-fluorinated dendrons.

We have reported similar effects of the fluorocarbon gas on the adsorption of a range of molecules, including phospholipids [36], polymers [17], proteins [37], biomarkers [38] and CeO_2_ nanoparticles [39]. But the most important finding here is that the fluorocarbon gas affects the adsorption of the IONPs differently, depending on whether the dendron carries a fluorinated end group or not. The interfacial tension at equilibrium (σ_eq_) of the dendronized IONP dispersions and characteristic times of adsorption (τ) of the latter for various Fe concentrations are collected in Table 1. The τ values were determined by fitting the adsorption profiles (Figure 3) to an exponential decay function. The variations of σ_eq_ versus Fe concentration are plotted in Figure 4a. The differences (Δσ_eq_) between σ_eq_ of dendronized IONPs exposed to air and those exposed to *F*-hexane-saturated air are plotted as a function of Fe concentration (Figure 4b). The Δσ_eq_ are larger for the fluorinated dendrons than for their hydrogenated analogs (7.0 ± 1.3 vs 1.1 ± 1.0 mN m^−1^).

These results indicate that in the presence of the fluorocarbon gas the surface excess of fluorinated dendrons is higher than for the hydrogenated analogs, or that the fluorinated dendrons form a more densely organized film at the interface. Either way, these results strongly suggest the existence of fluorous interactions between the end groups of the dendrons and the supernatant fluorocarbon gas that facilitate the adsorption of the IONPs at the interface. The mutual interactions between fluorinated chains are known to be weak, yet effective attractive interactions can operate in water and organic solvents. For example, such interactions are responsible for the partition and segregation of *F*-alkyl chains, on which “fluorous” technologies are based that are used in many synthesis and separation processes [40]. However, studies of such interactions published to date are restricted to liquid/liquid and solid/liquid interfaces [41–42]. In contrast, the potential of fluorocarbon gases to develop attractive fluorous interactions at the gas/water interface has only recently been demonstrated [38]. It is noteworthy that the interactions between fluorinated chains are reinforced by very effective hydrophobic repulsion caused by the proximity of the water phase.

**Figure 5:**
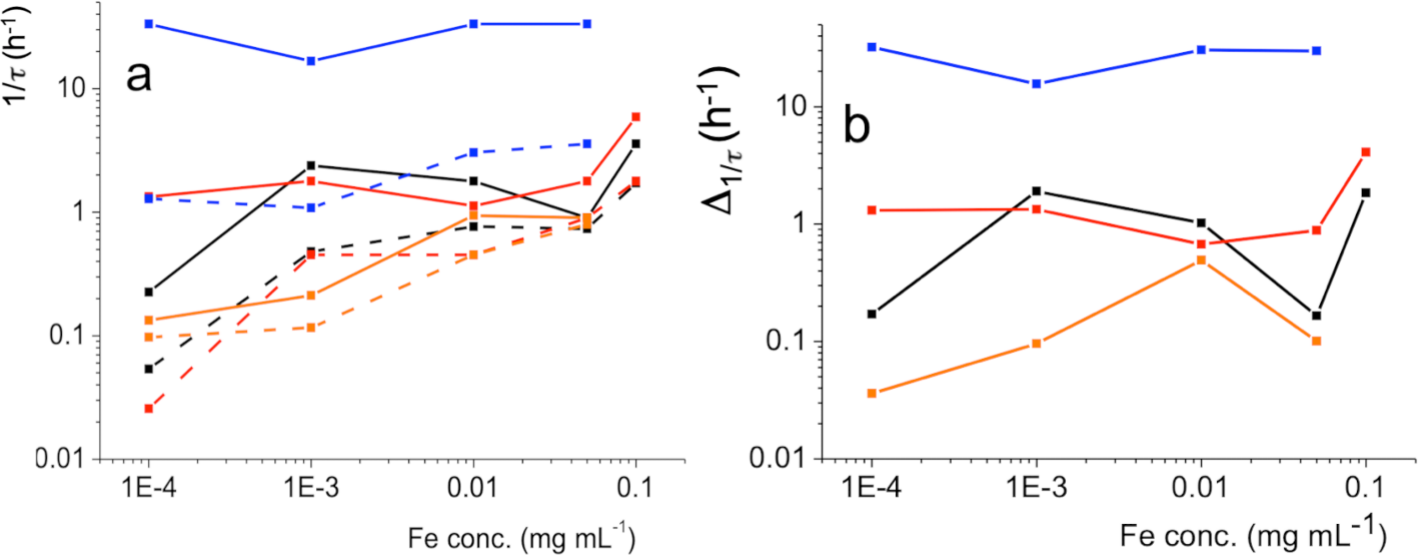
a) Variation of the inverse of the characteristic adsorption time (1/τ) of the IONPs grafted with dendrons (C_2_H_5_OEG_8_Den: black; C_2_F_5_OEG_8_Den: red; C_4_H_9_OEG_8_Den: orange; C_4_F_9_OEG_8_Den: blue) at the air (dashed) and *F*-hexane-saturated air (solid line)/aqueous phase interface. b) Variation of the differences of 1/τ measured under air and *F*-hexane-saturated air (Δ1/τ) as a function of the Fe concentration (same color code).

Figure 5 depicts the inverse of the characteristic adsorption time (1/τ) as a function of the Fe concentration of the dendronized IONPs. The 1/τ values increase with increasing Fe concentration (Figure 5a), except for the C_4_F_9_OEG_8_Den case, for which adsorption is only slightly increased (under air) or remains constant (under *F*-hexane). In all cases, the adsorption of the IONPs is accelerated by exposure to the *F*-hexane gas (solid lines). The magnitude of this effect depends on the degree of fluoration of the dendron. The differences between the 1/τ values measured under air and under *F*-hexane exposure (Δ1/τ) are collected for each dendronized IONP in Figure 5b. The largest Δ1/τ values are obtained for the dendron fitted with the C_4_F_9_ end group, which indicates that the strength of the interactions between *F*-hexane and the terminal group increases with the number of fluorinated carbons of the latter.

### Preparation and characterization of microbubbles incorporating dendronized iron oxide nanoparticles

**Figure 6:**
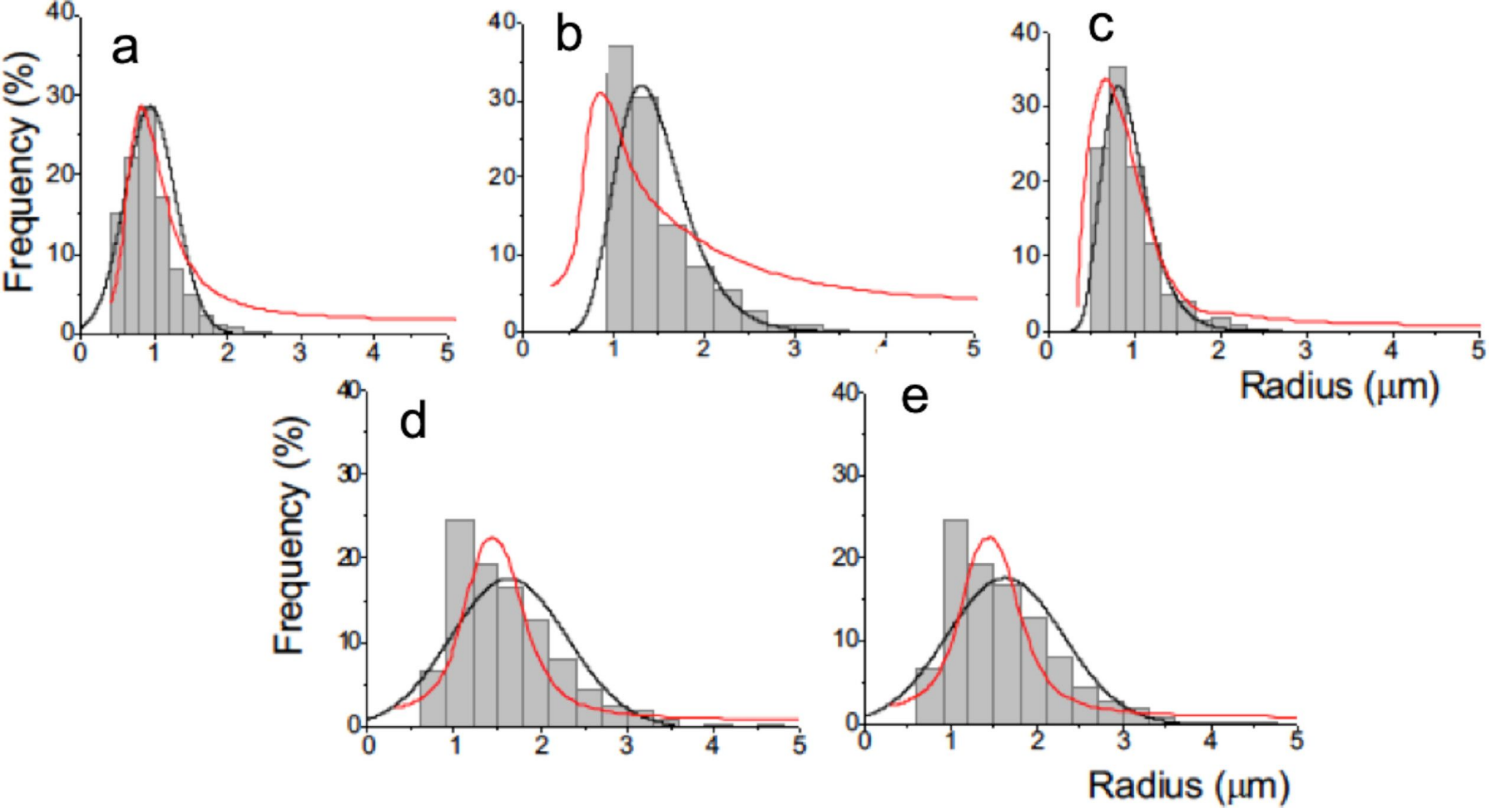
Size distributions of DPPC and DPPC/dendronized IONP-shelled microbubbles stabilized with *F*-hexane (grey line: Gaussian fit of the size histograms derived from optical microscopy; red line: distributions obtained by the acoustical method). a) DPPC alone; b–e) DPPC/IONP@C*_n_*X_2_*_n_*_+1_OEG_8_Den mixtures with b) C_2_H_5_OEG_8_Den; c) C_2_F_5_OEG_8_Den; d) C_4_H_9_OEG_8_Den, and e) C_4_F_9_OEG_8_Den. The concentration of IONPs was 0.1 mg mL^−1^; temperature 25 °C.

Our microbubbles were prepared by mixing aqueous dispersions of DPPC and dendronized IONPs conditioned in vials that have a dead volume saturated with *F*-hexane, in a VialMix shaker (Experimental section). The size and stability characteristics of the *F*-hexane-stabilized microbubbles prepared with DPPC and dendronized IONPs were investigated by optical microscopy and ultrasound wave attenuation analysis. MBs stabilized by a shell of DPPC were investigated for comparison. Our acoustic device measures the variation of the attenuation coefficient of an ultrasound wave as a function of its frequency at the initial measuring time in the measuring cell (Experimental section)_._ The change of the radius distributions over time is calculated from the attenuation curves. To this end, the bubble fraction is plotted against time allowing for the determination of the half-life of the bubbles [38]. The size and stability characteristics of the MBs incorporating the dendronized IONPs in their DPPC shell are provided in Table 2 and Figure 6.

**Table 2:** Physical characteristics of the DPPC microbubbles with dendronized IONPs, the mean bubble radius derived by optical microscopy (*R*_mean_ (optical, µm)), the bubble radius obtained by the acoustical method (Raman (acoustical, µm)) and the determined half-life of the bubbles (*t*_1/2_ (h)).

	DPPC	DPPC/ C_2_H_5_OEG_8_Den	DPPC/ C_2_F_5_OEG_8_Den	DPPC/ C_4_H_9_OEG_8_Den	DPPC/ C_4_F_9_OEG_8_Den

*R*_mean_ (optical, µm) *R*_mean_ (acoustical, µm) *t*_1/2_ (h)	0.9 ± 0.1 0.8 ± 0.2 6.8 ± 0.5	1.6 ± 0.2 1.1 ± 0.2 3.6 ± 0.7	1.0 ± 0.2 0.9 ± 0.2 6.1 ± 0.9	1.4 ± 0.1 1.4 ± 0.2 1.3 ± 0.2	1.0 ± 0.2 1.2 ± 0.2 5.0 ± 0.9

**Figure 7:**
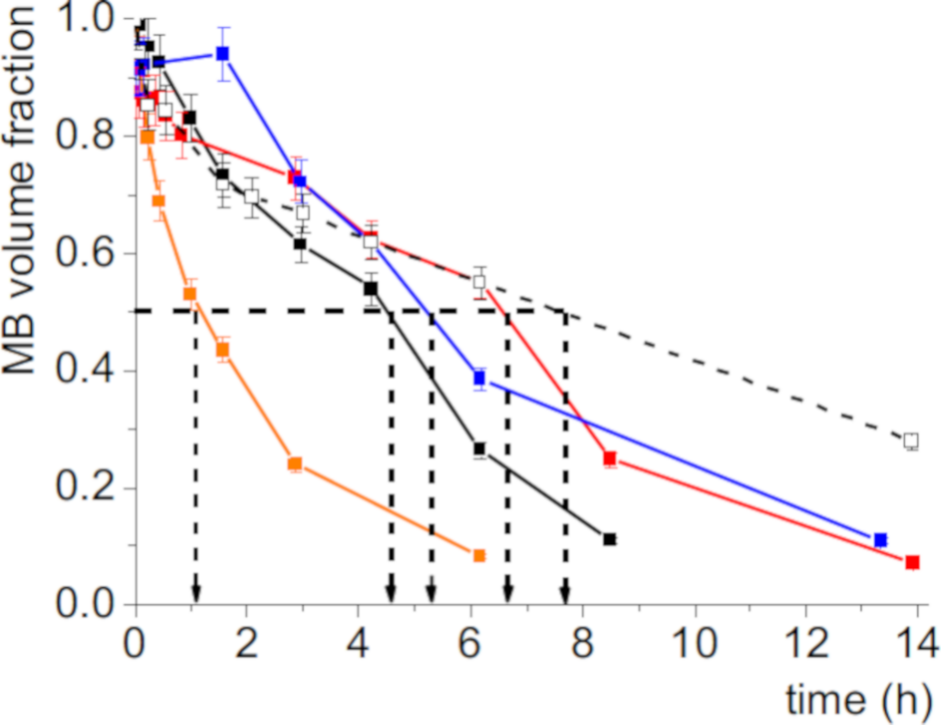
Time evolution (25 °C) of the volume fraction of the DPPC microbubbles (dotted grey) and of the DPPC microbubbles incorporating various IONPs: IONP@C_2_H_5_OEG_8_ (black); IONP@C_2_F_5_OEG_8_ (red); IONP@C_4_H_9_OEG_8_ (orange); IONP@C_4_F_9_OEG_8_ (blue).

The addition of dendronized IONPs led to a significant change in the MB mean radius and the size distribution for all the dendronized IONPs investigated, confirming their presence in the MB shell. A mean radius as small as 1.0 ± 0.2 µm was obtained with the fluorinated dendrons C_2_F_5_OEG_8_Den and C_4_F_9_OEG_8_Den, which is comparable to that measured for a non-dendronized DPPC shell. By comparison, the use of hydrogenated dendrons led to an increase in the MB mean radius. The stability of the MBs prepared with the fluorinated IONPs, given by the half-life of the corresponding bubbles, was also significantly higher than for those prepared with non-fluorinated NPs and, at least for C_2_F_5_OEG_8_Den (6.1 ± 0.9 h), comparable to that of DPPC (6.8 ± 0.5 h; Table 2 and Figure 7). These differences in behavior that depend on the fluorination of the dendron indicate that fluorous interactions exist between *F*-hexane in the gas core and the fluorinated NPs and play a significant role for the MB size and stability characteristics.

### AFM analysis of spin-coated films of DPPC, dendronized iron oxide nanoparticles and their mixtures

**Figure 8:**
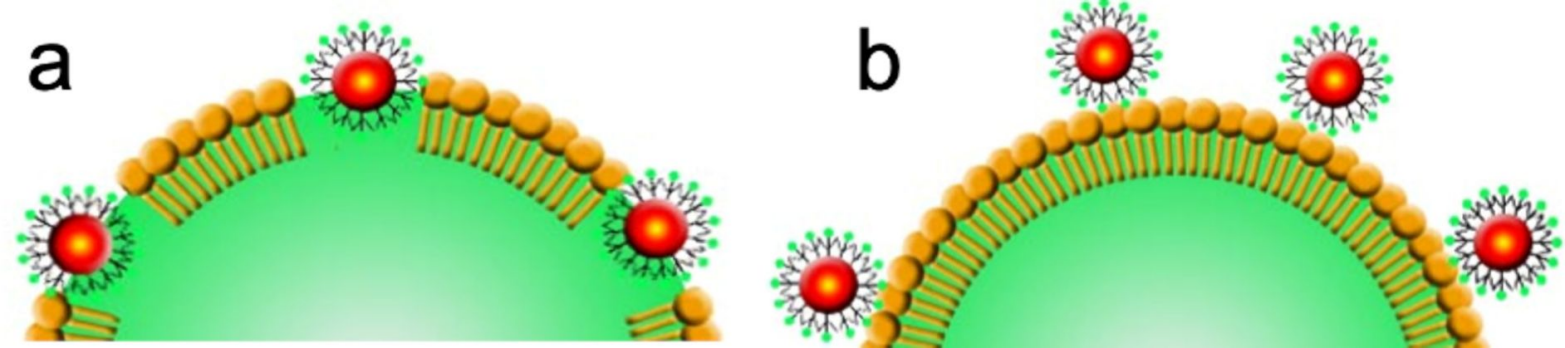
Schematic representation of dendronized IONPs a) incorporated within the MB DPPC shell and b) located at the surface of this shell.

With the aim to understand if the dendronized IONPs are incorporated within the DPPC shell of the MBs or located at the surface of the shell (Figure 8), mixed films composed of phospholipid and nanoparticles were prepared by spin-coating on silicon wafers. The morphology of the films was investigated by AFM in the peak–force tapping mode. We therefore selected C_2_F_5_OEG_8_Den, which is the dendron that led to the smallest and most stable MBs. The hydrocarbon analog C_2_H_5_OEG_8_Den was also investigated for comparison. The DPPC concentration was set in order to obtain a discontinuous DPPC film (i.e*.*, DPPC domains), allowing for the measurement of the film height.

**Figure 9:**
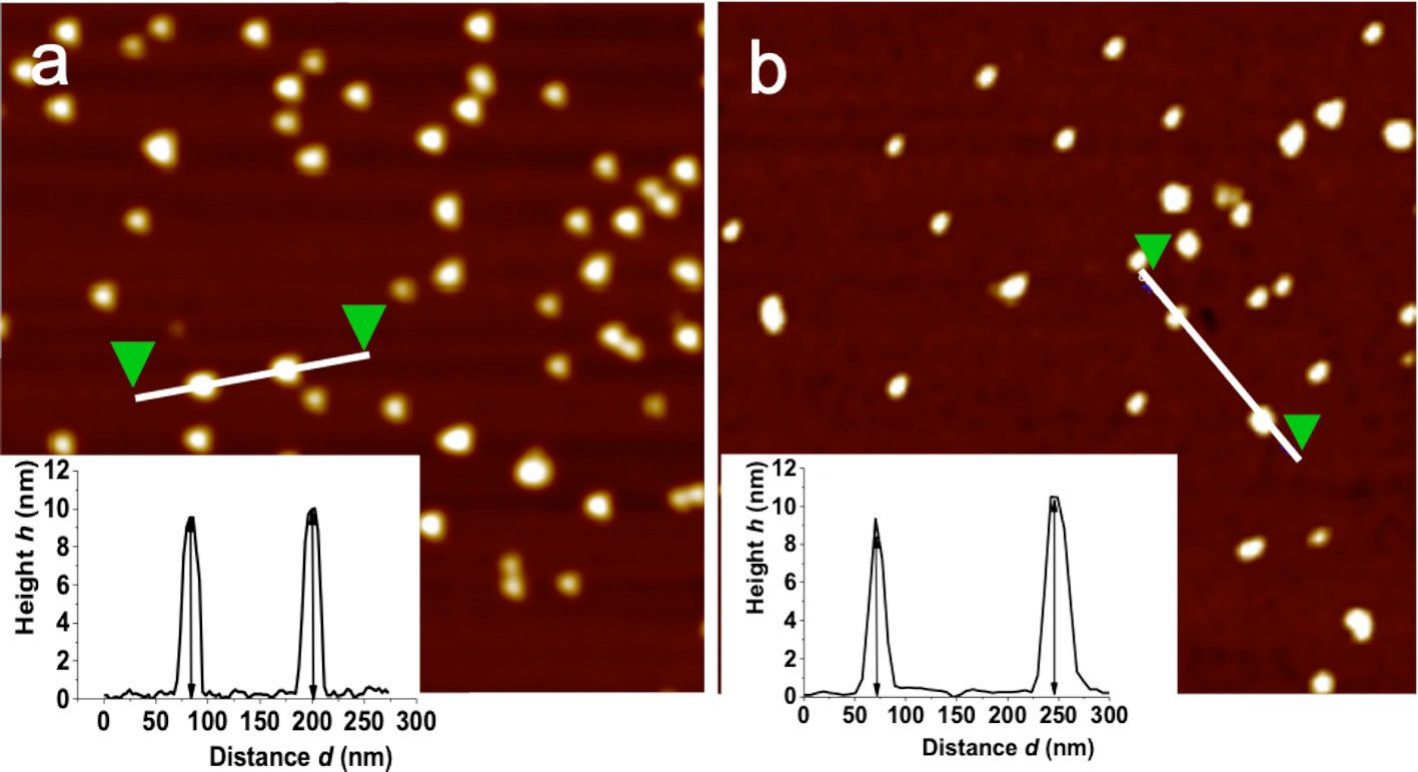
AFM topography images (1 × 1 µm) and height profiles of a) IONP@C_2_F_5_OEG_8_Den and b) IONP@C_2_H_5_OEG_8_Den. Dispersions of IONPs in ethanol (0.002 mg mL^−1^) were spin-coated on silicon wafers.

**Figure 10:**
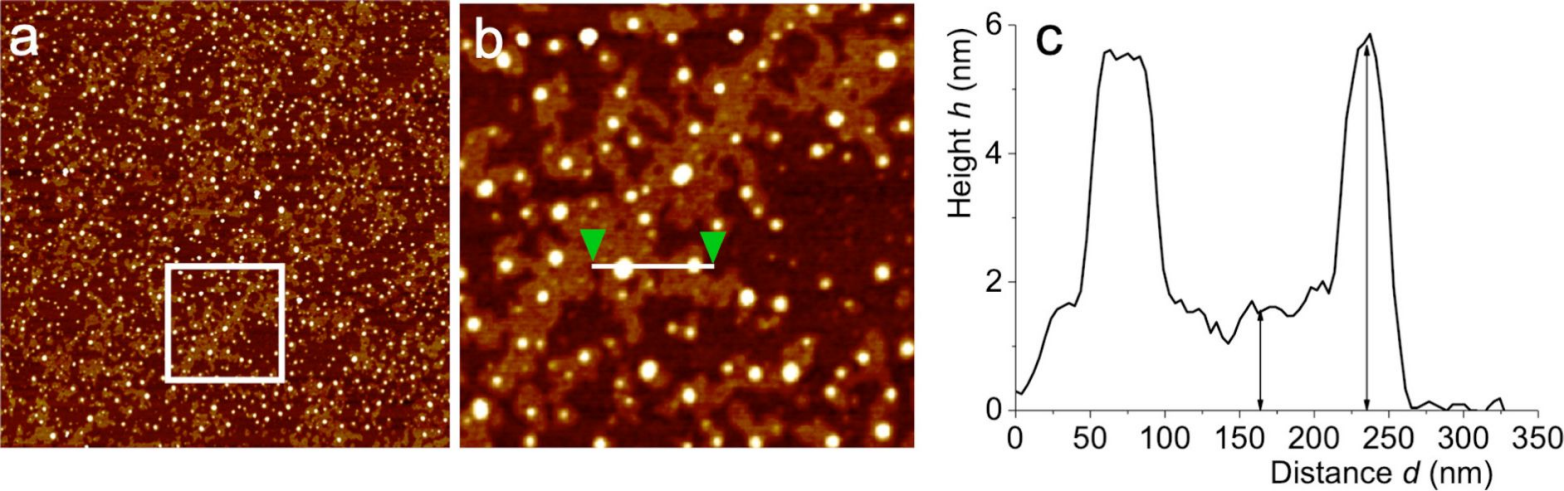
a) AFM topography image (4 × 4 µm) of a DPPC film spin-coated from an ethanol solution (0.5 mM); b) magnification (1 × 1 µm) of the square shown in a); c) height profile taken between the two green triangles in b).

The mean height of both IONP@C_2_F_5_OEG_8_Den and IONP@C_2_H_5_OEG_8_Den is 10.0 ± 1.7 nm, as determined by a statistical analysis of the particles (Figure 9). Usually, it is observed that the nanoparticles are convoluted by the AFM probe, which decreases the lateral resolution of the technique. Both IONP samples are well-dispersed with no indication of aggregation. The films of spin-coated DPPC form large monolayer and small bilayer domains (Figure 10a). The profile measured on the magnified image (Figure 10b and Figure 10c) shows that the heights of the monolayer and bilayer are 1.5 ± 0.3 nm and 5.0 ± 1.0 nm, respectively. These measurements are in agreement with earlier reports [43].

**Figure 11:**
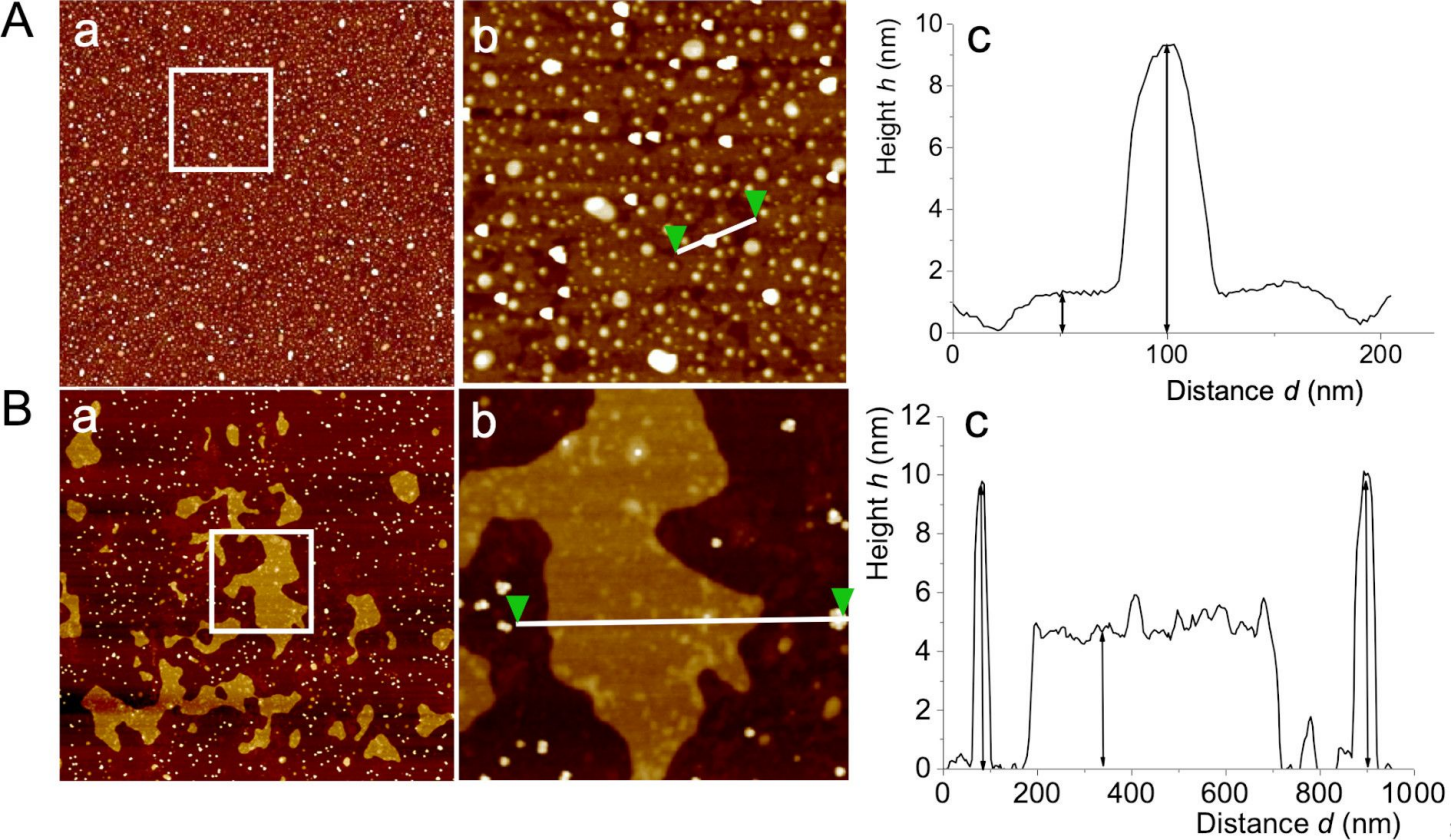
a) AFM topography image (4 × 4 µm) of the mixed spin-coated films composed of DPPC and IONP@C_2_F_5_OEG_8_Den (Panel A) and IONP@C_2_H_5_OEG_8_Den (Panel B); b) magnification images (1 × 1 µm) of the white square shown in a); c) height profiles taken between the two green triangles in the corresponding image in b). Co-dispersions of DPPC (0.5 mM) and IONPs (0.002 mg mL^−1^) in ethanol were spin-coated on silicon wafers.

Figure 11A shows an AFM topography image of a mixed film composed of DPPC and IONP@C_2_F_5_OEG_8_Den. The IONPs are embedded within the DPPC monolayer domains in which they are well-dispersed, showing no tendency to aggregate. The profile (Figure 11Ac) measured on the magnified image (Figure 11Ab) clearly shows that the fluorinated IONPs are incorporated into the 1.5 nm thick DPPC monolayer. A different morphology is observed for the mixed film of DPPC and IONPs grafted with the hydrogenated dendron C_2_H_5_OEG_8_Den. In this case, the domains formed by DPPC bilayers of ≈5 nm in height are omnipresent, while only a few domains of monolayers are observed (Figure 11Ba). It is seen that IONP@C_2_H_5_OEG_8_Den are preferentially located in the regions of the wafer that are devoid of phospholipid domains. The height profile measured on the magnification image (Figure 11Bb) shows two IONPs of ≈10 nm in height, clearly separated by a bilayer domain of ≈5 nm in height.

This difference can be explained by the fact that short fluorinated groups such as C_2_F_5_ can significantly increase the lipophilicity of molecules. This is the main reason why fluorine groups, such as CF_3_ or C_2_F_5_, are incorporated into many drugs, as they significantly improve their biodistribution [44]. By contrast, longer fluorinated chains, such as C_6_F_13_ or C_8_F_17_, are well-known to confer a lipophobic character when grafted onto molecules and to induce phase separation in fluorocarbon/hydrocarbon mixtures [45–46]. These results tell us that the dendronized IONPs fitted with a C_2_F_5_ group have a higher affinity for the phospholipid film than those grafted with C_2_H_5_.

## Conclusion

We report that small and highly stable magnetic MBs incorporating IONPs in their phospholipid shells can be obtained by using IONPs dendronized with OEG chains. The latter significantly increase the dispersibility of the nanoparticles in aqueous media.

We demonstrate that exposure to a supernatant fluorocarbon gas has a remarkable and considerable impact on the adsorption behavior of dendronized iron oxide nanoparticles and that the magnitude of this effect depends on the nature of the end group of the dendron grafted on the nanoparticles, which is either fluorinated or hydrogenated. Introducing a short fluorinated group at the end of the OEG chain is found to substantially increase the rate of adsorption of the nanoparticles at the interface with air and even more so when exposed to *F*-hexane-saturated air. A more compact film is observed when the film of nanoparticles is exposed to the fluorocarbon gas. This unexpected effect indicates that for the mixed film, at the interface, interactions develop between the fluorinated end group of the dendron and the fluorocarbon gas.

As a consequence of this new phenomenon, small and stable fluorocarbon-stabilized microbubbles with a half-life of ≈6 h can be obtained by admixing DPPC and iron oxide nanoparticles that are grafted with a C_2_F_5_-terminated dendron. The combined use of fluorinated dendrons and a supernatant fluorocarbon gas is a straightforward, effective method for preparing magnetic microbubbles that could facilitate the development of future applications in medicine.

Finally, the AFM analysis of the DPPC/iron oxide nanoparticles films indicates that the fluorinated dendronized iron oxide nanoparticles show a higher propensity to incorporate into phospholipid films than into hydrogenated ones, possibly due to the increased lipophilic character.

## Experimental

### Materials

1,2-Dipalmitoylphosphatidylcholine (DPPC) was purchased as a dry powder (99% purity) from Avanti Polar Lipids (Alabaster, AL) and used as received. Perfluorohexane (98% pure) was purchased from Fluorochem. Pluronic F-68 (a poly(ethylene oxide)−poly(propylene oxide) triblock copolymer, *M*_W_≈8300, purity >99%) and HEPES (*N*-(2-hydroxyethyl)piperazine-*N*′-2-ethanesulfonic acid) were purchased from Sigma-Aldrich (Lyon, France). A HEPES buffer solution (20 mmol L^−1^) in a 150 mmol L^−1^ NaCl solution was prepared, and its pH was adjusted to 7.4 with 1 N NaOH. Water was purified using a Millipore system (surface tension 71.4 mN m^−1^ at 20 °C, resistivity 18.2 MΩ cm).

### Synthesis of dendrons

**Scheme 1:**
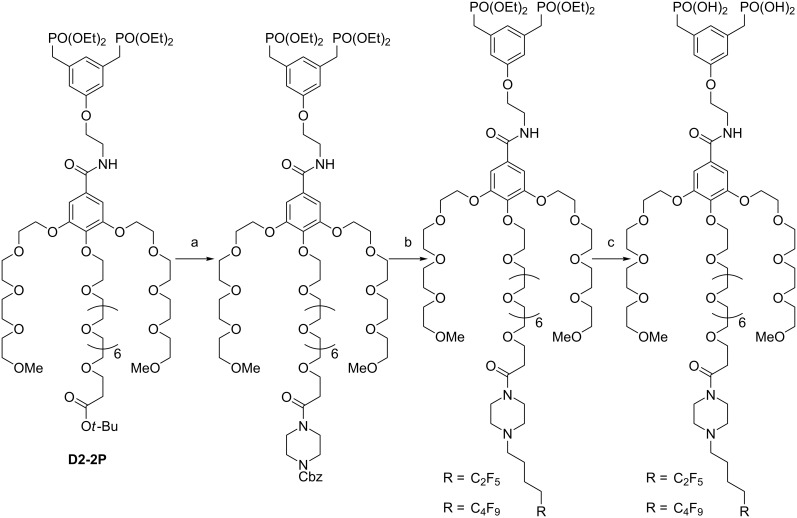
Final steps of the synthesis of the dendrons C_2_F_5_OEG_8_Den and C_4_F_9_OEG_8_Den. a) TFA/CH_2_Cl_2_, then piperazine-NCBz, HATU, DIPEA/DMF; 99% (2 steps); b) H_2_, Pd/C/ MeOH, then perfluoroalkyl iodide, K_2_CO_3_/CH_3_CN; 80% for C_2_F_5_ and 55% for C_4_F_9_; c) TMSBr/CH_2_Cl_2_; 79% for C_2_F_5_ and 80% for C_4_F_9_.

The approach to the synthesis of the dendrons C_2_F_5_OEG_8_Den and C_4_F_9_OEG_8_Den is described in [47]. From the intermediate D2-2P, the piperazine unit was installed in two steps (Scheme 1): 1) deprotection of the *tert*-butyl group and 2) amide coupling by using HATU/DIPEA. Next, the removal of the carboxybenzyl group by hydrogenolysis allows for the introduction of the perfluoroalkyl chain via *N*-alkylation. Finally, treatment with trimethylsilyl bromide produced the desired fluorinated bisphosphonate dendron.

Characterization of C_2_F_5_OEG_8_Den: ^1^H NMR (500 MHz, CD_3_OD) δ 7.25 (s, 2H), 6.84 (s, 1H), 6.79 (s, 2H), 4.22 (t, *J* = 4.5 Hz, 6H), 4.15 (t, *J* = 5.5 Hz, 2H), 3.87 (t, *J* = 4.7 Hz, 4H), 3.80 (t, *J* = 4.7 Hz, 2H), 3.76–3.50 (m, 52H), 3.33 (s, 6H), 3.12–3.09 (m, 2H), 3.03 (d, *^2^J_P-H_* = 21.1 Hz, 4H), 2.65–2.62 (m, 2H), 2.27–2.16 (m, 2H), 1.88–1.82 (m, 2H), 1.68–1.62 (m, 2H) ppm; ^13^C NMR (125 MHz, CD_3_OD) δ 172.3, 169.5, 160.1, 153.8, 142.4, 136.6, 130.5, 115.3, 107.8, 73.6, 73.0, 71.9, 71.7–71.3 (several peaks), 70.8, 70.1, 68.5, 67.4, 62.2, 59.1, 57.3, 41.0, 39.6, 34.2, 24.2 ppm; ^19^F NMR (282 MHz, CD_3_OD) δ −86.9, −119.4 ppm; ^31^P NMR (202 MHz, CD_3_OD) δ 22.7 ppm.

Characterization of C_4_F_9_OEG_8_Den: ^1^H NMR (500 MHz, CD_3_OD) δ 7.25 (s, 2H), 6.83 (s, 1H), 6.78 (s, 2H), 4.22 (t, *J* = 4.5 Hz, 6H), 4.14 (t, *J* = 5.6 Hz, 2H), 3.87 (t, *J* = 4.7 Hz, 4H), 3.80 (t, *J* = 4.6 Hz, 2H), 3.76–3.50 (m, 74H), 3.33 (s, 6H), 3.10–3.06 (m, 2H), 3.02 (d, *^2^J_P-H_* = 21.0 Hz, 4H), 2.64–2.59 (m, 2H), 2.32–2.21 (m, 2H), 1.89–1.83 (m, 2H), 1.70–1.63 (m, 2H) ppm; ^13^C NMR (125 MHz, CD_3_OD) δ 172.3, 169.5, 160.1, 153.8, 142.4, 136.6, 130.5, 125.3, 115.3, 108.9, 79.3, 73.6, 73.0, 72.1, 71.9–71.3 (several peaks), 70.8, 70.0, 68.5, 67.4, 62.2, 59.1, 57.3, 52.9, 52.5, 43.7, 41.0, 39.6, 34.2, 30.8, 24.2, 18.7 ppm; ^19^F NMR (470 MHz, CD_3_OD) δ −82.6, −115.6, −125.3, −127.1 ppm; ^31^P NMR (202 MHz, CD_3_OD) δ 22.4 ppm.

### Synthesis of dendronized iron oxide nanoparticles

The synthesis is adapted from an earlier report [33]. In a 100 mL two-necked flask, iron(II) stearate (2.2 mmol, 1.38 g), oleic acid (4.4 mmol, 1.24 g) and dioctyl ether (20 mL) were mixed together. The resulting solution was heated to 120 °C for 1 h under magnetic stirring without a reflux condenser. The magnetic stirrer was removed and the condenser was connected to the flask. The solution was heated up to 298 °C for 2 h at a heating rate of 5 °C min^−1^. After cooling, a black suspension was collected and precipitated by addition of acetone. Finally, the nanoparticles were washed three times with a mixture of CHCl_3_/acetone (1:4). In a 30 mL vial, a nanoparticle suspension in tetrahydrofuran (THF) (5 mg of iron) was introduced together with the appropriate dendron (7 mg). The vial was filled with 25 mL of THF and the mixture was magnetically stirred for 24 h. The resulting nanoparticles were centrifuged after addition of cyclohexane, dispersed in water and separated by ultrafiltration. The grafting of the fluorinated end-group was evidenced using HR-MAS.

### Bubble profile analysis tensiometry

Axisymmetric bubble shape analysis was applied to a rising bubble of gas (air or *F*-hexane-saturated air) formed in a dispersion of dendronized IONPs in an aqueous phase (HEPES buffer). As described in [36], during the process of adsorption of the dendronized IONPs at the gas/liquid interface, the variation of the interfacial tension was acquired using a Tracker^®^ tensiometer (Teclis, Civrieux d’Azergues, France). A 5 µL bubble was formed at the end of a steel capillary that had a tip diameter of 1 mm. The rising bubble was saturated with *F-*hexane by purging a 1 mL syringe trice with *F*-hexane-saturated air sampled above liquid *F*-hexane. This syringe was then mounted immediately on the injection cell of the tensiometer, such that the rising bubble was formed. The pressure and concentration of the *F*-hexane-saturated vapor at 25 °C were set to 2.9 × 10^4^ Pa and 11.66 mol m^−3^ [14]. IONP dispersions with Fe concentrations ranging from 0.1 to 10^−4^ mg mL^−1^ were obtained by diluting the 1 mg mL^−1^-concentrated stem aqueous dispersions with HEPES buffer. The IONP@C_4_X_9_OEG_8_Den (X = F and H) aqueous dispersions were sonicated for 30 min (setting 5) before tensiometric measurement. The sonicator (Vibracell, Bioblock Scientific, Illkirch, France) was equipped with a 3 mm titanium probe and operated at 20 kHz with an output power of ≈600 W (duty cycle 40%).

### Preparation of the microbubbles

DPPC (50 mmol L^−1^) and Pluronic F-68 (DPPC/F-68 molar ratio 10:1) were dispersed in a non-degassed HEPES buffer solution (0.9 mL) in a sealed glass vial (inner diameter of 13 mm, length of 35 mm) by magnetic stirring for 3–6 h at 50 °C. Pluronic F-68 was added to facilitate phospholipid dispersion and foam formation. 100 µL of the dendronized IONPs dispersion (Fe concentration of 1 mg mL^−1^) were injected into the dispersion. The dispersions were sonicated under air in a sonication bath at 50 °C for 30 min. In the case of IONP@C_4_X_9_OEG_8_Den (X = H or F), presonication (2 min, setting 5) under air was applied. N_2_ was allowed to bubble through three vials containing *F*-hexane before being flushed above the aqueous phase into the sealed glass vial during 3 min in order to saturate the gas phase with *F*-hexane. The resulting dispersions were treated using a VialMix shaker (Bristol-Myers Squibb, New York, NY) for 45 s under *F*-hexane-saturated N_2_ at room temperature. The resulting foam was immediately diluted to 10 mL of HEPES buffer. Size fractionation of the microbubbles was achieved by flotation for 60 min.

### Optical microscopy

A few droplets (three to four) of the bubble dispersion were positioned in a concave glass slide and covered with a glass slide. The samples were observed using a Nikon Eclipse 90i microscope (transmission mode). Rapid image acquisition was obtained with a Lumenera Infinity 2 charge-coupled device (CCD) camera (Lumenera, Ottawa, Canada). The mean radii of the bubbles were determined using ImageJ on 5−10 slides.

### Acoustic size determination

The method exploits the sound attenuation undergone by multifrequency ultrasound waves that propagate through the aqueous bubble dispersion. Standard simple-harmonic resonator curves are fitted to measure the attenuation in order to infer the radii of the bubbles. A low-power emitter is used to avoid alteration of the bubble characteristics and stability. For further experimental details see [48]. Each measurement was repeated three times for different bubble preparations. The volume of the microbubble dispersion injected in the acoustic cell was 2 mL.

### AFM topography analysis of mixed films of DPPC and dendronized IONPs

Thin films of DPPC, dendronized IONPs and DPPC/dendronized IONP mixtures were prepared by spin-coating on silicon wafers [49]. To this end, a dispersion of dendronized IONPs in water (1 mg L^−1^) was freeze-dried and then dissolved in ethanol for preparing a dispersion with a concentration of 0.1 mg mL^−1^. 40 µL of this dispersion was added to 2 mL of a 1 mM-concentrated DPPC ethanol solution in order to obtain a mixed DPPC/dendronized IONP spin-coated film that has the same DPPC/Fe molar ratio as that used for the preparation of the microbubbles (28:1). A 0.5 mM-concentrated mixed dispersion was obtained by diluting this 1 mM dispersion. Silicon wafers were cleaned for 30 min in a sonication bath containing ethanol/milli-Q water (1:1), followed by 2 min in a plasma cleaner. A droplet (15 µL) of DPPC, dendronized IONPs or mixed DPPC/dendronized IONP dispersions was deposited on a silicon wafer and immediately spun for 1 min at 3000 rpm (Spin150 from SPS, Semiconductor Production Systems Europe). The spin-coated samples were placed under vacuum in a desiccator for 15‒20 h to fully evaporate the solvents. The silicon wafers were stored at 4 °C until the AFM measurements. AFM images were obtained by scanning the spin-coated films using a Dimension AFM Icon (Bruker) instrument operated in peak–force tapping mode. Peak–force AFM is based on the peak–force tapping technology, in which the probe is oscillated in a similar way as in the tapping mode, but at far lower resonance frequency. Each time the tip and the sample are brought together, a force curve is captured. Ultrasharp silicon tips on a nitride lever were used (Bruker, ScanAsyst with a spring constant of 0.4 N m^−1^ and tip radius of ≈5 nm). During AFM imaging, the force was reduced in order to avoid dragging of molecules by the tip. The analysis of the images was conducted in the integrated software. At least three different samples were analyzed and several positions were scanned on the silicon wafer for each sample. The error on measurements along the *z*-axis was estimated at ±0.5 nm.

## Supporting Information

File 1 Additional spectra.
