# Supplementary material for: Microbubbles decorated with dendronized magnetic nanoparticles for biomedical imaging: effective stabilization via fluorous interactions
Source: Beilstein J Nanotechnol. 2019 Oct 31;10:2103–15. doi: 10.3762/bjnano.10.205 (PMC6839566; doi:10.3762/bjnano.10.205)
Supplement: File 1 — Additional spectra. [file Beilstein_J_Nanotechnol-10-2103-s001.pdf]

BEILSTEIN JOURNAL OF NANOTECHNOLOGY

Supporting Information

for

Microbubbles decorated with dendronized magnetic nanoparticles for biomedical imaging: effective stabilization via fluorous interactions

Da Shi, Justine Wallyn, Dinh-Vu Nguyen, Francis Perton, Delphine Felder-Flesch, Sylvie Bégin-Colin, Mounir Maaloum and Marie Pierre Krafft

Beilstein J. Nanotechnol. **2019,** *10,* 2103–2115. doi:10.3762/bjnano.10.205

Additional spectra

License and Terms: This is a supporting information file under the terms of the Creative Commons Attribution License (http://creativecommons.org/licenses/by/4.0). Please note that the reuse, redistribution and reproduction in particular requires that the authors and source are credited. The license is subject to the *Beilstein Journal of Nanotechnology* terms and conditions: (https://www.beilstein-journals.org/bjnano)

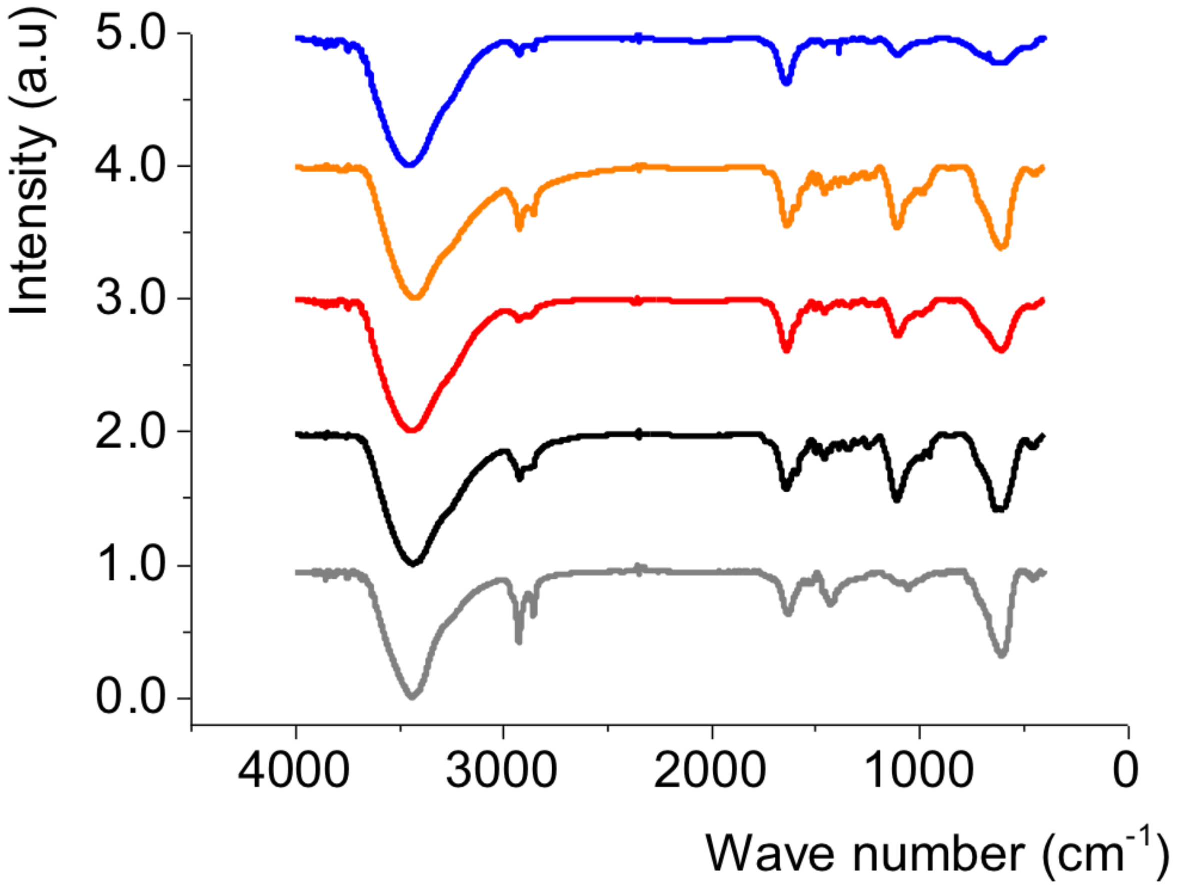

Figure S1: Infrared spectra of IONPs covered by oleic acid (grey), or dendronized with $C_2H_5OEG_8Den$ (black), $C_2F_5OEG_8Den$ (red), $C_4H_9OEG_8Den$ (orange) and $C_4F_9OEG_8Den$ (blue).

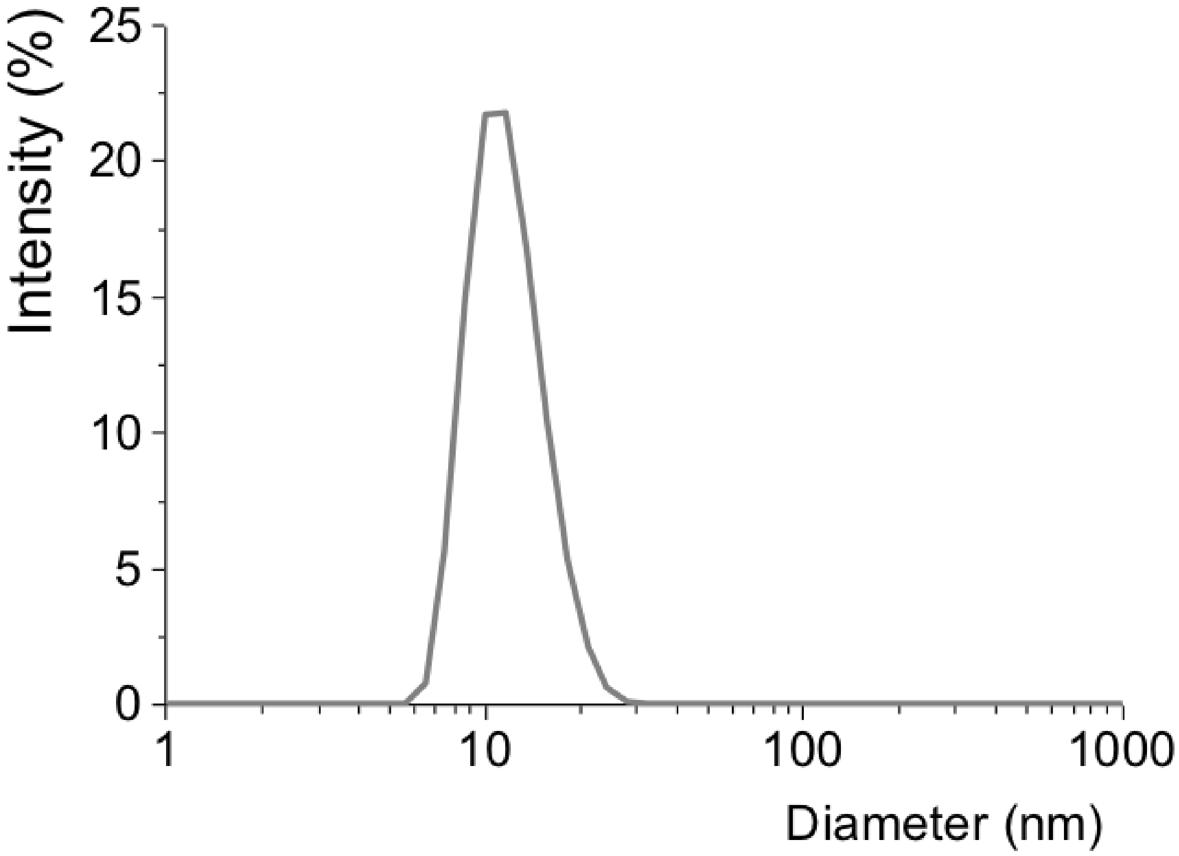

Figure S2: Hydrodynamic diameter distributions of IONPs covered with oleic acid (10 ± 1 nm), in suspension in THF aqueous dispersions (0.05 mg mL^{-1}).

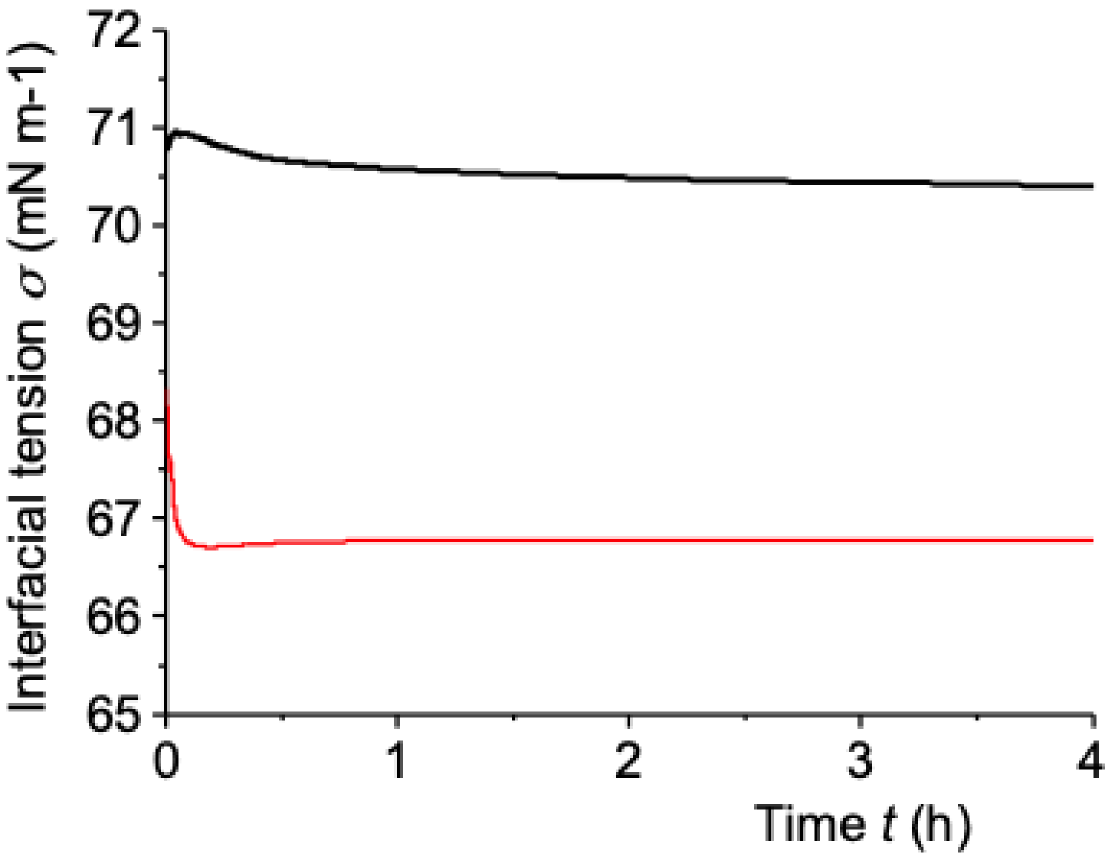

Figure S3: Variation of the interfacial tension σ of the gas/aqueous phase interface as a function of time under air (black) and under *F*-hexane-saturated air (red). Temperature was 25 °C.
